# Delayed Splenic Rupture 48 Hours After a Colonoscopy: A Case Report

**DOI:** 10.7759/cureus.101763

**Published:** 2026-01-18

**Authors:** Parthena Samara, Sergios Konstantinidis, Anastasios Katsourakis, Michael N Chrysikos, Konstantinos Peidis, Iosif Hadjis

**Affiliations:** 1 General Surgery, General Hospital of Thessaloniki "Gennimatas-Agios Dimitrios", Thessaloniki, GRC; 2 Urology, General Hospital of Thessaloniki "Gennimatas-Agios Dimitrios", Thessaloniki, GRC

**Keywords:** acute abdomen, colonoscopy, complication, hemoperitoneum, splenectomy, splenic rupture

## Abstract

Splenic rupture following colonoscopy, although uncommon, is a potentially life-threatening complication that all medical doctors should consider in their differential diagnosis. Several risk factors contribute to this condition, including exogenous elements such as the endoscopist's experience, patient positioning, medications, mechanical force during endoscope advancement, and technique at the splenic flexure, as well as endogenous factors like patient anatomy, perisplenic adhesions, inflammatory or infectious diseases, hematological disorders, malignancy, and interventions such as biopsy or polypectomy. We report a case of a 59-year-old woman who presented to the emergency department 48 hours after a colonoscopy with left upper quadrant abdominal pain, weakness, and pallor. Laboratory tests showed anemia. She had ongoing pain and hemodynamic instability, and a computed tomography revealed hemoperitoneum caused by splenic rupture. The patient underwent splenectomy and subsequently recovered without complications. Although colonoscopy remains the gold standard for diagnosing and managing colorectal diseases with a low incidence of serious adverse events, physicians should maintain a high index of suspicion for splenic injury in patients presenting with anemia and recent large bowel endoscopy.

## Introduction

Approximately six million screening colonoscopies are performed annually in the United States. However, about 15 million colonoscopies are conducted each year for various purposes, including screening, surveillance, and other specific indications. A systematic analysis of the studies conducted on screening colonoscopies between 2002 and 2019 was carried out by Fraiman et al. [1] and included roughly 450,000 patients. Their review highlighted the overuse of screening colonoscopy in the population that was either older or younger than those recommended by the US national guidelines, thereby exposing them to unjustified risk.

On par with any minimally invasive procedure, a screening colonoscopy might encompass certain complications; however, the benefits far exceed the risks [1,2]. Reumkens et al., in their meta-analysis comparing the post-colonoscopy complication rate over the 15 years between 2001 and 2015, concluded that this rate remained stable or even declined due to the monitoring after the procedure [3]. The overall complication rate is estimated to be 0.5%, with hemorrhage and bowel perforation being the most prevalent complications. By contrast, splenic injury remains rather uncommon, with an incidence rate of only 0.001% [4,5]. Patients experiencing a splenic rupture most frequently describe abdominal pain that is either non-localized or limited to the left upper quadrant and radiating to the lumbar region or the left shoulder (Kehr sign) within 24 hours of the procedure [6]. Other minor presentations include dizziness, syncope, and symptoms of anemia [7].

Here, we report a case of a woman in her late 50s who presented to the emergency department with abdominal pain. After a series of diagnostic tests, she was finally diagnosed with splenic rupture as a result of colonoscopy that had been performed 48 hours prior to the beginning of her symptoms. The aims of this paper are to highlight the significance of reviewing a patient's medical history when neither a clinical examination nor blood tests can provide conclusive information and to raise concern regarding this underreported complication.

## Case presentation

A 59-year-old female patient with prior appendectomy and no comorbidities, hematological diseases or history of trauma presented to the emergency department with complaints of abdominal pain localized in the left upper quadrant, tenderness to the costovertebral angle, and fatigue for the past 48 hours. She alerted us to an intravenous (IV) contrast media allergy. She made no mention of any previous procedure in the past few days.

On arrival, the patient was hemodynamically normal, with a pulse of 60 beats/min, blood pressure 103/60 mmHg, and no signs of hypovolemia. Our examination revealed left abdominal tenderness to palpation with mild to moderate involuntary guarding, the absence of a rebound sign, a positive Giordano sign on the left side, and a negative Kehr’s sign. A digital rectal examination was negative for blood.

Laboratory results revealed anemia, with hemoglobin levels up to 8.93 g/dL (normal range: 11.8-17.8 g/dL), hematocrit 27.3 (normal range: 37.7-47.9%), and white blood cells within the normal range of 10.2 × 10³/μL (normal range: 4.9-10.8 × 10³/μL), of which 63.4% were neutrophils. The rest of the inflammatory indicators were slightly over the upper limit of normalcy (CRP=0.56 mg/dL; normal range: 0-0.5 mg/dL) (Table 1).

**Table 1:** Laboratory tests

Laboratory Test	Patient's result	Normal reference range
Hemoglobin (g/dL)	8.93	11.8-17.8
Hematocrit (%)	27.3	37.7-47.9
White blood cells (× 10³/μL)	10.2	4.9-10.8
Neutrophils (%)	63.4	~40-70%
C-reactive protein (mg/dL)	0.56	0-0.5

Urinalysis did not reveal anything abnormal.

**Figure 1:**
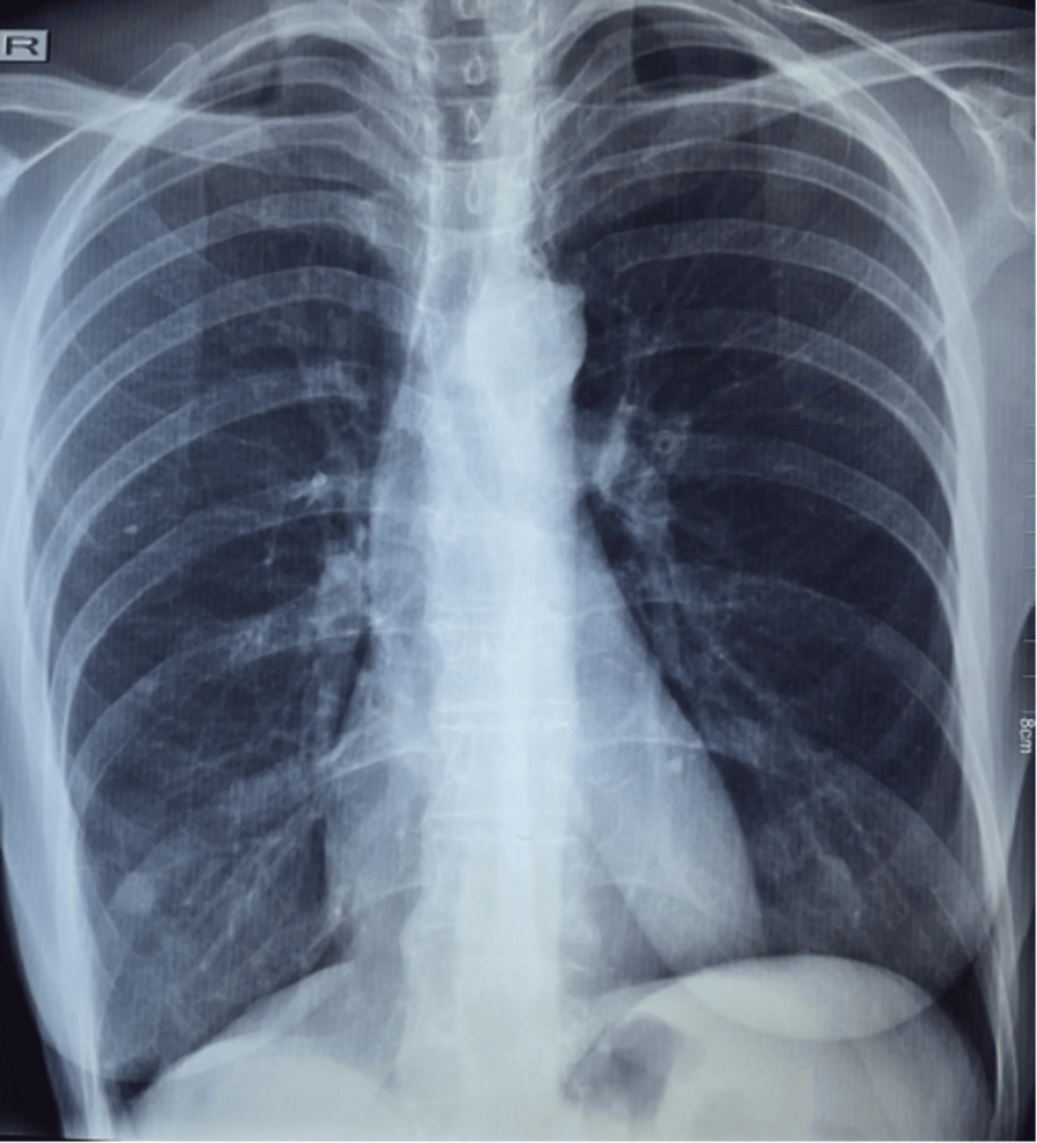
Abdominal X–ray showing the absence of pneumoperitoneum

The chest X-ray (Figure 1) was negative for air under the diaphragm, and an abdominal X-ray was not indicative of ureteric stones.

She was admitted to the hospital for further investigation. Approximately six hours after admission (roughly 54 hours post-colonoscopy), the patient experienced an episode of loss of consciousness, and she became hemodynamically unstable, with a drop in blood pressure to 70/40 mmHg and a increase of heart rate to 130 beats/min. A repeat complete blood count (CBC) revealed a critical drop in hemoglobin to 5.41 g/dL representing a decline of 3.52 g/dL from her baseline emergency room laboratory exams. Intravenous fluids were initiated, along with red blood cell transfusion (three packs), fresh frozen plasma (two packs) and broad- spectrum antibiotic was started (cefoxitin). A focused assessment with sonography in trauma (FAST) ultrasound showed fluid around the hepatorenal recess and the perisplenic area. During the ultrasound examination, the patient mentioned a screening colonoscopy two days before, that had been omitted from her original medical history. According to the colonoscopy report that was later on obtained, the position used was a left lateral one, the Boston-Bowel-Preparation-Scale was eight (RC2, TC3, LC3), advancing through the splenic flexure had difficulties but normal force was applied overall, and a polyp of approximately 0.5 centimeters in diameter located in the descending colon was removed with endoscopic biopsy forceps. Spasms and loops were present at the sigmoid colon, third-degree hemorrhoids were diagnosed and the endoscope was removed approximately after nine minutes.

**Figure 2:**
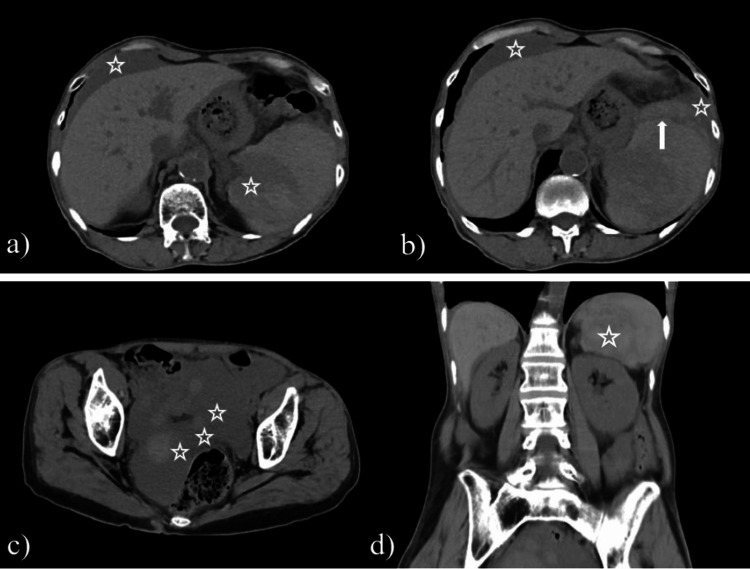
CT scan images a) Markers indicate hemoperitoneum around the liver and the spleen; b) Arrow indicates the location of the rupture and star markers indicate hemoperitoneum around the liver and spleen; c) Hyperdense free fluid in the Douglas Pouch; d) Coronal image, star marker indicates the location of the ruptured spleen and the surrounding perisplenic hematoma.

A computed tomography (CT) scan without IV or oral contrast media was performed to resolve the broad range of differential diagnoses correlated with complications after colonoscopy and to establish the necessity of an emergency surgery. The images revealed an enlarged, inhomogeneous spleen with a significant amount of hyperdense fluid around it, indicating active bleeding and a large peritoneal effusion between the intestinal loops, around the liver and the spleen related to hemoperitoneum (Figure 2).

The patient underwent an emergency exploratory laparotomy that revealed a grade V [8] splenic laceration and hemoperitoneum. Upon accessing the peritoneal cavity, hemoperitoneum was drained. During the laparotomy, extensive peritoneal adhesions were observed among the patient’s organs and structures, which were unexpected given the patient’s limited surgical history (surgical history of appendectomy with a McBurney incision). Mobilization of the spleen was achieved and a total splenectomy was performed following the sequential ligation of the splenic vessels. The specimens were sent for histological evaluation and no histopathological abnormalities were observed.

The patient had an uneventful recovery and was discharged five days later with instructions for a standard post-splenectomy vaccination and a hemoglobin level of 10 mg/dL and a hematocrit of 29.2%. At the time of re-examination at the outpatient department, the patient adduced a CT scan report of upper and lower abdomen as well as of the retroperitoneal area that was performed approximately three months prior to the colonoscopy without any pathological findings of importance regarding our case.

## Discussion

The spleen is the largest organ of the lymphatic system and is positioned in the left hypochondriac region between the ninth and eleventh ribs [9]. Splenic injuries are divided into two broad categories: atraumatic and traumatic. Recognized as a common complication during abdominal surgery, iatrogenic splenic rupture is estimated to be responsible for up to 40% of all splenectomies [10]. A wide range of surgeries, including upper gastrointestinal procedures, colectomies, left nephrectomy, and vascular operations involving the abdominal aorta, entail a high risk of intraoperative splenic injury. Yet, according to Feola et al., colonoscopy is responsible for the majority of iatrogenically induced splenic injuries [11]. Nevertheless, splenic rupture following colonoscopy is quite an atypical complication, with a currently unknown incidence rate, given the limited number of case reports in the published literature. Kamath et al. [5] reviewed 296,248 patients who underwent colonoscopy in their institution from 1980 to 2008 and reported that only four developed a splenic injury, corresponding to an incidence rate of 0.001%. However, considering the rising number of colonoscopies performed annually, extremely rare complications like splenic injuries could potentially be reported more frequently since roughly 1.3% of the patients who undergo the procedure will visit the emergency department within a week with various complaints [12]. In a literature review that was conducted in 2009, 81% out of the 66 patients presented in the emergency department complaining about abdominal pain within 48 hours of the procedure and over a third of the patients described left upper quadrant pain [13].

The predominant mechanism for splenic injury following colonoscopy is tension on the splenocolic ligament; however, we noted considerable pathological adhesions during laparotomy. We hypothesize that these adhesions were the main culprit for the injury, since they seemed to constrain the splenic flexure, as it established a fixed point of resistance. The advancement of the colonoscope likely transmitted tension directly to the splenic parenchyma due to the reduced mobility of the colon, resulting in a tear of the capsule. This traction led to a subcapsular hematoma that remained encapsulated until its eventual rupture after 48 hours later [14]. Conventional endoscopic training emphasizes the seamless navigation of the splenic flexure to avoid excessive tension on the splenocolic ligaments. Nonetheless, the presence of firm peritoneal adhesions, as evidenced in this case, might constrain the colon, significantly increasing the risk of damage despite the use of proper technique.

Epidemiological research by Piccolo et al. [15] reviewed 103 cases, and indicated that female sex and a history of abdominal surgery were important risk factors for splenic injury. However, a detailed analysis of our patient's clinical progression suggested that these factors demonstrated their influence in a different manner. The patient's female sex could have established a fundamental anatomical vulnerability, such as a potentially deeper splenic flexure or mobile ligament. Nevertheless, we consider the intraoperative recognition of dense peritoneal adhesions to be the most significant contributing factor.

Additional risk factors include polypectomy during colonoscopy, anticoagulant treatment, splenomegaly or conditions that may develop abdominal adhesions such as history of trauma, laparotomy, inflammatory bowel disease, and pancreatitis [15]. Most patients with speculated splenic injury experience acute abdominal pain combined with Kehr’s sign and low hemoglobin levels within 24 hours after the procedure except for rare cases that have delayed presentation up to 10 days after. The imaging of choice in such cases is CT, as it precisely delineates the diagnosis and the presence of active bleeding [16].

The exact etiology of delayed splenic rupture is not known, but there is a hypothesis. A subcapsular hematoma takes place and due to the increased oncotic pressure because of cell lysis it ruptures. Another suggestion of the mechanism of action can be the formation of a pseudoaneurysm [17,18].

The management of splenic injury could either be conservative or surgical, depending on the severity of the injury, as described in the literature. Hemodynamically stable patients with low grade injuries (I-III) are preferably managed conservatively with intravenous fluid replacement, hemoglobin-hematocrit monitoring or embolization. On the contrary, unstable patients or patients with higher grade injuries (>IV) and active bleeding are managed with emergency laparotomy and splenectomy. A systematic review of the literature from 2002 to 2010 by Corcillo et al. [19] compared the three types of treatment (conservative, arterial embolization or splenectomy) performed during splenic injuries after colonoscopy and reported that 74% of the patients eventually underwent laparotomy and splenectomy. More specifically, out of the 77 patients reviewed, conservative treatment was performed in 25 (32.5%) of the patients but rebleeding occurred in 11 out of the 25, so they eventually underwent surgery. Lastly, 7.8% of the patients were managed with arterial embolization. The authors suggested that, even in cases where active bleeding is present, less invasive treatment is more beneficial. 

## Conclusions

Splenic rupture, although rare, presents an important threat after colonoscopy, requiring meticulous evaluation, especially when patients display vague abdominal symptoms within the latent period following the procedure. This case report highlights the potential of extensive peritoneal adhesions as a mechanical factor for delayed splenic rupture leading to rapid clinical deterioration and severe splenic trauma (Grade V). While modern methods like arterial embolization offer less invasive treatment alternatives for stable patients, in patients with substantial clinical deterioration, exploratory laparotomy serves as the conclusive, life-saving procedure.
